# Effect of inoculation with prion dilutions within the dynamic range of ELISA absorbance on prion incubation period

**DOI:** 10.1007/s11259-022-10013-w

**Published:** 2022-10-11

**Authors:** Eric D. Cassmann, Quazetta L. Brown, Alexis J. Frese, Zoe J. Lambert, M. Heather West Greenlee, Justin J. Greenlee

**Affiliations:** 1 United States Department of Agriculture, Virus and Prion Research Unit, National Animal Disease Center, Agricultural Research Service, Ames, IA USA; 2 Oak Ridge Institute for Science and Education, 1299 Bethel Valley Rd., Oak Ridge, TN 37830 USA; 3 Department of Biomedical Sciences, Iowa State University College of Veterinary Medicine, 1800 Christensen, Ames, IA 50011 USA

**Keywords:** Scrapie, Incubation, ELISA, Titration, Mouse, TSE, Prion

## Abstract

This study examines the effect of various infectious prion titers within the dynamic range as measured by ELISA on incubation period. We inoculated ovinized transgenic mice with seven decreasing dilutions of a fast-incubating scrapie strain. The highest inoculum group was a 20% w/v brain homogenate from a sheep with scrapie. The subsequent six inoculum dilutions ranged from the highest ELISA optical density reading of 4.000 to a dilution where scrapie prions were not detectable by ELISA. Multiple comparison analysis demonstrated variation in the incubation periods between some inoculum groups. Incubation periods were similar between inoculum groups unless their optical density differed by more than ≈2 units of absorbance. These data will inform the interpretation of future studies that compare incubation periods in experimentally inoculated animals for TSE research.

## Short communication

Transmissible spongiform encephalopathies (TSEs), also referred to as prion diseases, are a group of fatal neurodegenerative diseases caused by a misfolded form (designated as PrP^Sc^) of the prion protein (Prusiner 1998). There are several species-specific TSEs affecting non-human animals including scrapie in sheep and goats (Prusiner 1998), bovine spongiform encephalopathy (mad cow disease) in cattle (Wells et al. 1987), and chronic wasting disease in cervids (Williams and Young 1992). Due to the protracted incubation period of TSEs in their natural hosts, experiments are frequently carried-out in mouse models.

The incubation period of TSEs is influenced by host-recipient genetics, TSE strain, age, and infectious dose (Bruce 2003; Ryder et al. 2009). A meta-analysis of 117 dose titration studies compared the relationship between infectious dose and incubation period in mice (McLean and Bostock 2000). The studies applied broad log10 dose- titrations to examine variability in incubation periods. The authors of the meta-analysis observed that incubation period increases linearly relative to a logarithmic decrease in dose, and incubation period variability is greater at lower doses.

Enzyme linked immunoassays (ELISAs) are frequently used to confirm a TSE diagnosis. In the present study, we sought to determine the variability in incubation period relative to doses within the dynamic range of ELISA absorbances. In the context of this assay, the dynamic range was defined as the range of dilutions extending from the lowest point on curve to the highest (4.000 OD). The results shed light on how the dose titer of infectious prions within the dynamic range of ELISA absorbances influences incubation period, and will assist in the comparison of incubation periods of inoculated animals for future TSE research.

**Fig. 1:**
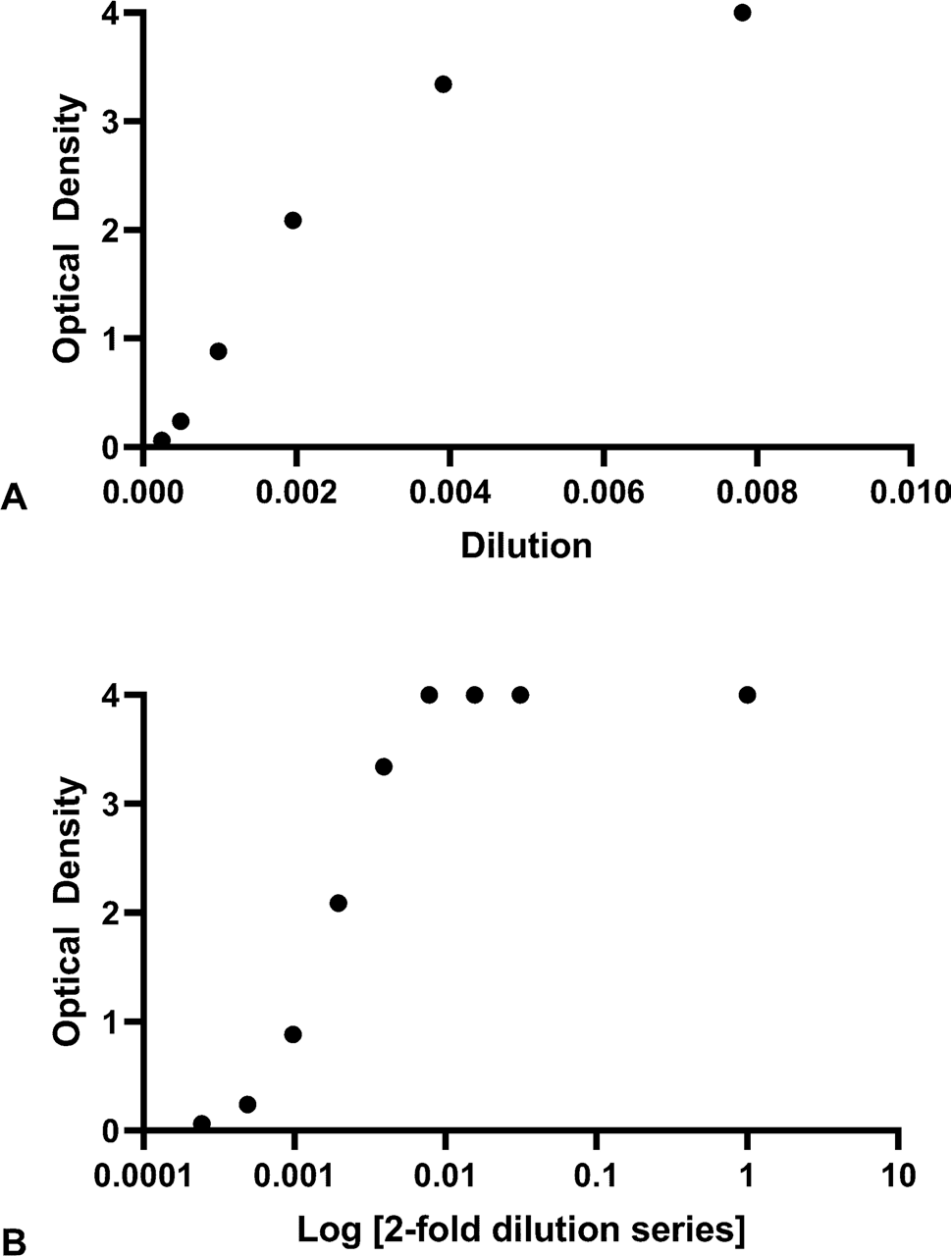
Determination of the ELISA dynamic range for the inocula. (**A**) Optical density on the y-axis plotted against a 2-fold dilution series ranging from 4096x (1/4096 = 0.000244) to 128x (1/128 = 0.00781) left-to-right. (**B**) Log transformed 2-fold dilutions corresponding to 4096x, 2048x, 1024x, 512x, 256x, 128x, 64x, 32x, and 20% w/v homogenate compared to optical density on the y-axis left-to-right

To determine the necessary dilutions to obtain optical densities between 4.0 (max absorbance) and undetectable scrapie prions, we used a 20% w/v brain homogenate from a Suffolk sheep (VRQ/VRQ) that had been inoculated with the brain pool homogenate from the ×124 fast incubating scrapie strain (Bulgin et al. 2006; Hamir et al. 2009; Moore et al. 2016) to perform a series of dilutions. The stock brain homogenate had an OD of 4.000 and was considered to have quantities of PrP^Sc^ vastly greater than what would be expected for a maximum prion concentration to achieve an OD of 4.000. Therefore, we initially started with a 32x dilution and proceeded with a 2x dilution series until the optical density dropped below 4.000. All dilutions were made with phosphate buffered saline. The last dilution to result as a 4.000 optical density (128x) was considered a true 4.000 reading. Subsequent dilutions corresponded closely with whole number optical densities until the final dilution resulted in *non-detectable* PrP^Sc^ (Table 1 and Fig. 1).

**Table 1:** Inocula dilutions with corresponding ELISA results and resulting bioassay incubation periods

Sample dilution factor	ELISA O.D.	Test result	n° mice^a^	Mean IP (days)	Std. deviation	Median IP (days)
20% w/v homogenate	4.000	POS	12	66.5	4.462	68
32x	4.000	POS	b	b	b	b
64x	4.000	POS	b	b	b	b
128x	4.000	POS	13	88.9	3.87	89
256x	3.342	POS	13	90.5	4.858	91
512x	2.088	POS	15	94.7	6.914	92
1024x	0.880	POS	17	102.4	10.36	100
2048x	0.237	POS	16	102.5	4.633	102
4096x	0.063	ND	14	106.3	5.497	108

^a^Euthanized mice included in the calculation of incubation period were ELISA positive (100% attack rate); ^b^ no animal groups were inoculated for the 32x and 64x dilutions; IP, incubation period; POS, positive; ND, non-detect; ELISA cut-off, 0.201

To examine the variation in incubation periods between different ELISA optical densities, we intracranially inoculated 20 uL of brain homogenate into cohorts of ovinized transgenic (Tg338) mice (Vilotte et al. 2001) based on the different sample dilutions. Seven groups of mice were inoculated with either the stock homogenate (undiluted, denoted 1x) 20% w/v, 128-, 256-, 512-, 1024-, 2048-, or 4096-fold dilutions. Mice were monitored daily for clinical signs associated with prion disease in mice including poor hygiene/haircoat, ataxia, circling, or inability to right position. Upon observation of clinical signs, mice were humanely euthanized, and brains were collected for ELISA (HerdChek; IDEXX Laboratories, Westbrook, ME) to confirm prion disease. Mice that died prior to observation of clinical signs were not used to calculate the incubation period even though they were ELISA positive for prion disease; this was to ensure an accurate measurement of time between inoculation and clinical disease onset (incubation period). The final data set included 12, 13, 13, 15, 17, 16 and 14 mice from each group, respectively. The mean incubation periods corresponding to each inoculum group were 66.5, 88.9, 90.5, 94.7, 102.4, 102.5, and 106.3 days (Table 1).

**Fig. 2:**
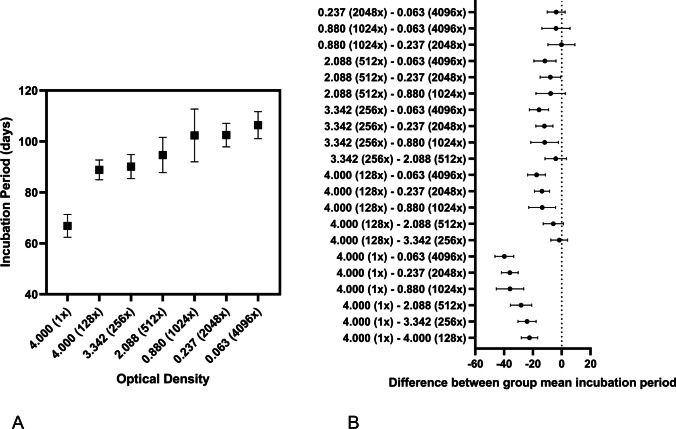
Comparison of ELISA adjusted samples to incubation period. (**A**) Mean incubation period in days on the y-axis plotted against the dilution group and corresponding optical density (error bars indicate standard deviation). (**B**) Results from Dunnett’s T3 multiple comparisons analysis. Error bars overlapping zero difference between group means indicates no significant difference between groups (α = 0.95)

To assess differences between incubation periods in each inoculum group, we performed Brown-Forsythe and Welch ANOVA tests followed by Dunnett’s T3 multiple comparisons test using GraphPad Prism version 9.3.1 for Windows, GraphPad Software, San Diego, California USA, www.graphpad.com (Fig. 2). The results of the ANOVA tests showed a significant difference between groups (p < 0.0001). For multiple comparisons, the greatest differences in incubation periods were noted between the 20% w/v inoculum group (1x) and all other optical density adjusted dilutions (p < 0.0001). Differences in the incubation period were not significantly different for seven optical densities comparisons: 4.0 vs. 3.342, 4.0 vs. 2.088, 3.342 vs. 2.088, 2.088 vs. 0.880, 0.880 vs. 0.237, 0.880 vs. 0.063, and 0.237 vs. 0.063 (Fig. 2B).

Our results demonstrate that prion titers within the measurable optical density range influence the incubation period in our model. However, differences in incubation period are less substantial and frequently insignificant when both optical densities are above or below ≈2.000. For example, the difference in incubation periods between 3.342 and 2.088 was not statistically significant, but the difference between 3.342 and 0.880 was significant. This caveat does not hold true for samples with 4.000 values exceeding the maximum absorbance reading of 4.000. The titer of infectious prions in a 20% w/v brain homogenate was 128-fold above the maximum ELISA absorbance, and inoculation of mice resulted in a significantly shorter incubation period compared to an optical density of 4.000 where it drops into the dynamic absorbance range. An interesting observation was that mice inoculated with the ELISA *negative* 4096x dilution had similar incubation periods to positive ELISA optical densities of 0.880 and 0.237. This is not a novel discovery; instead, it simply underscores the superior sensitivity of mouse bioassays and cautions interpretation of ELISA results as *negative*. A better designation for non-positive ELISA results is non-detectable. Given that previous mouse studies of prion titers demonstrated differences in attack rates and incubation periods after several logarithmic dilutions of sample (Andreoletti et al. 2011; Douet et al. 2014; McLean and Bostock 2000), we did not expect there to be a significant difference in incubation periods within the measurable range of ELISA optical densities that corresponded to 128-fold to 4096-fold dilutions. However, results from this study suggest that differences greater than ≈2 units of absorbance within the ELISA measurable range and large titers exceeding the maximum OD of 4.000 can significantly influence the incubation period in mice. These findings will assist in the comparison of incubation periods of inoculated animals for future TSE research.

## Data Availability

The datasets generated during and/or analyzed during the current study are available from the corresponding author on reasonable request.
